# Evolution of sideways locomotion in crabs

**DOI:** 10.7554/eLife.110015

**Published:** 2026-08-27

**Authors:** Junya Taniguchi, Tsubasa Inoue, Kano Kohara, Jung-Fu Huang, Atsushi Hirai, Nobuaki Mizumoto, Fumio Takeshita, Yuuki Kawabata

**Affiliations:** 1 Graduate School of Integrated Science and Technology, Nagasaki University Nagasaki Japan; 2 National Kaohsiung University of Science and Technology Kaohsiung Taiwan; 3 Susami Crustacean Aquarium Wakayama Japan; 4 Department of Entomology and Plant Pathology, Auburn University Auburn United States; 5 Kitakyushu Museum of Natural History and Human History Kitakyushu Japan; Graduate University for Advanced Studies, SOKENDAI Japan; University of Neuchâtel Switzerland

**Keywords:** Brachyura, ancestral state reconstruction, key innovation, behavioral evolution, forward locomotion, evolutionary reversal, Other

## Abstract

The evolutionary change in the mode of locomotion is often a major evolutionary event, triggering diversification. Sideways locomotion is a defining feature of true crabs (Brachyura) and may have contributed to their ecological success. Yet, the evolutionary origin of this unique behavior remains unknown. Here, we show that the prevalence of sideways locomotion in true crabs reflects a single evolutionary origin from a forward-moving ancestor. Our behavioral analysis of 50 live crab species indicates that crab locomotion can be broadly separated into two predominant modes, sideways and forward locomotion. The phylogenetic comparative analysis revealed a single origin of sideways locomotion, with multiple independent reversions to forward locomotion in ecologically specialized groups. The species richness data show that the lineage in which sideways locomotion originated is far more diverse than its nearest outgroups. These results are consistent with the idea that sideways locomotion acted as a key innovation contributing to the evolutionary diversification of true crabs. Such a rare but innovative behavioral trait provides a framework for understanding how locomotor modes shape evolutionary diversification in animals.

## Introduction

The evolution of novel functional traits can enable organisms to exploit previously inaccessible ecological niches (Stroud and Losos, 2016; Miller et al., 2023). Such key innovations have shaped biodiversity on Earth by facilitating adaptive radiation within lineages. Because locomotion is a fundamental behavior that is involved in most survival and reproductive processes (Alexander, 2003; Domenici, 2010), innovations in locomotor mechanisms are often linked to adaptive radiations (Astudillo-Clavijo et al., 2015; Higham et al., 2015; Burress and Wainwright, 2019; Hedrick et al., 2020; Feiner et al., 2021). For example, the evolution of flight in insects opened aerial niches globally and contributed to their enormous radiation (Grimaldi and Engel, 2005), whereas modifications of the locomotor skeleton in *Anolis* lizards facilitated localized adaptive radiations within islands (Feiner et al., 2021). Most comparative studies on such innovations in locomotor behavior have taken a functional morphological approach. However, despite the recognized importance of behavioral aspects of key innovations (Miller et al., 2023), the actual locomotor behaviors have rarely been compared across species, largely due to the challenges of obtaining large, comparative datasets of animal behaviors.

True crabs (Infraorder Brachyura) are iconic for their sideways locomotion, enabling them to achieve fast bidirectional movements (Vidal-Gadea et al., 2008), which may be beneficial for escaping from predators (Wolfe et al., 2021). Sideways locomotion is associated with greater joint flexibility in the lateral direction and a thorax elongated along the preferred direction of locomotion (Vidal-Gadea et al., 2008). This unique locomotion could be a key innovation in decapod crustaceans, as it changes the behavioral axis through which animals interact with the environment (Miller, 1949). Moreover, sideways locomotion could have contributed to the ecological success of true crabs. The number of species of true crabs (~7904 species) far exceeds that of their sister group, Anomura (hermit crabs and others; ~3437 species), or their closest relatives, Astacidea (clawed lobsters and crayfish; ~792 species) and Achelata (spiny and slipper lobsters; ~153 species), according to the World List of Decapoda, DecaNet (DecaNet eds, 2025). True crabs have also successfully colonized diverse habitats globally, including terrestrial, freshwater, and deep-sea environments (Wolfe et al., 2024; DecaNet eds, 2025). In addition, the crab-like body plan has evolved repeatedly among decapod crustaceans, a phenomenon known as carcinization (Morrison et al., 2002; Tsang et al., 2011; Keiler et al., 2017; Wolfe et al., 2021). Despite this rich diversity and extensive morphological information, however, data on actual locomotor behaviors of crabs are sparse, and no comparative studies based on large datasets have been conducted thus far, making it difficult to evaluate the role of this unique locomotor mode on crab evolution and diversity.

Although most true crabs use sideways locomotion, some groups—including raninids, majids, and mictyrids—move predominantly forward (Sleinis and Silvey, 1980; Faulkes, 2006; Vidal-Gadea et al., 2008). This raises key questions: when did sideways locomotion originate, how many times did it evolve, and how many times did it revert? With a recent comprehensive crab phylogeny based on genomic data (Wolfe et al., 2024), here, we conducted behavioral analyses of 50 live crab species. We aimed to (i) pinpoint the origin of the sideways locomotion within Brachyura, (ii) estimate the number of transitions and reversions between sideways and forward locomotion, and (iii) test whether the emergence of sideways locomotion is associated with species diversification. Our results highlight sideways locomotion as a rare but innovative behavioral trait, providing a framework to understand how locomotor modes shape evolutionary diversification in animals.

## Results

**Figure 1.**
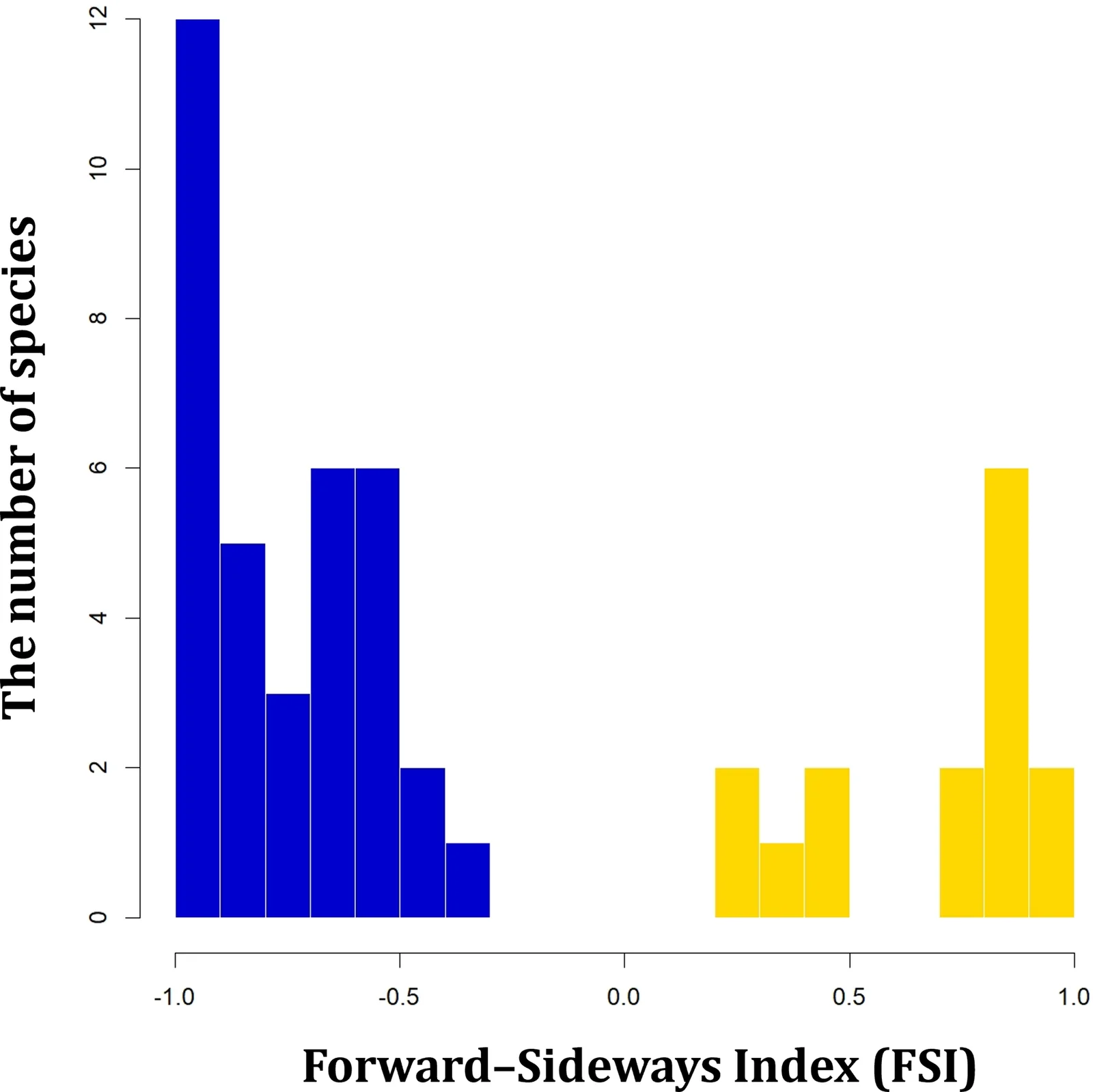
Distribution of Forward–Sideways Index (FSI) values among crab species exhibiting forward and sideways locomotion. Gold bars represent species classified as forward movers, and blue bars represent species classified as sideways movers. Figure 1—source data 1.Species-level Forward–Sideways Index (FSI) values and selected reference-circle radii for the 50 species analyzed.

**Figure 2.**
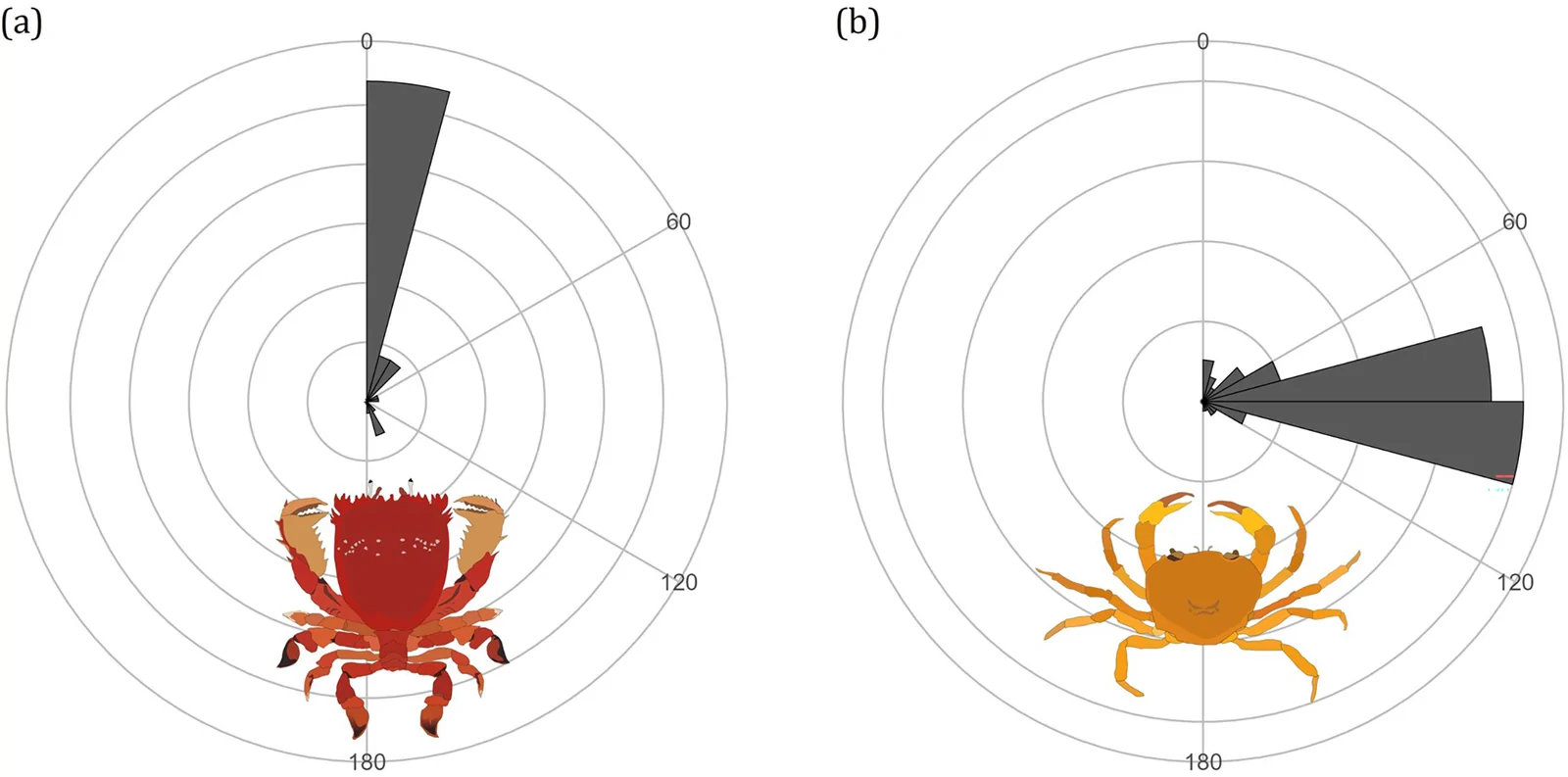
Representative circular histograms of movement directions in crabs. (**a**) Forward movement in *Ranina ranina* (FSI = 0.89). (**b**) Sideways movement in *Geothelphusa dehaani* (FSI = –0.70). The 0°–180° axis denotes the crab’s body axis before movement, with bars indicating the frequency of movement direction. Figure 2—source data 1.Tracking coordinates of the anterior and posterior carapace landmarks of *Ranina ranina* used to generate the circular histogram in Figure 2a. Figure 2—source data 2.Tracking coordinates from the first recording segment of *Geothelphusa dehaani* used to generate the circular histogram in Figure 2b. Figure 2—source data 3.Tracking coordinates from the second recording segment of *Geothelphusa dehaani* used to generate the circular histogram in Figure 2b. Figure 2—source data 4.Tracking coordinates from the third recording segment of *Geothelphusa dehaani* used to generate the circular histogram in Figure 2b. Figure 2—source data 5.Tracking coordinates from the fourth recording segment of *Geothelphusa dehaani* used to generate the circular histogram in Figure 2b. Figure 2—source code 1.R code used to calculate movement directions from tracking coordinates and generate the circular histogram for *Ranina ranina* in Figure 2b. Figure 2—source code 2.R code used to calculate movement directions from tracking coordinates and generate the circular histogram for *Geothelphusa dehaani* in Figure 2b.

We quantified locomotor direction using the Forward–Sideways Index (FSI), which ranges from −1 (completely sideways) to +1 (completely forward); species with FSI <0 were classified as sideways movers and those with FSI >0 as forward movers. Of the 50 species, 35 were classified as sideways movers and 15 as forward movers based on this index (Figure 1, Figure 1—source data 1). For example, *Ranina ranina* showed an FSI of 0.89, indicating forward movement, whereas *Geothelphusa dehaani* showed an FSI of –0.70, indicating sideways movement (Figure 2). FSI values showed a clear separation between these locomotion modes: forward movers had a median FSI of 0.82 (range: 0.24 to 0.94), while sideways movers had a median FSI of –0.80 (range: –1.00 to –0.39) (Figure 1). This separation was supported by Hartigan’s dip test (*D*=0.083, n=50, p=0.007), indicating significant deviation from unimodality. All species-level circular histograms are provided in Figure 2—figure supplements 1–3. To further examine the underlying angle distributions, we fitted one- and two-component Gaussian mixture models to the continuous bout-angle distributions of each taxon and examined peak locations and mixture weights. In total, 14 taxa were best described by a one-component model, whereas 36 taxa were best described by a two-component model. Among the 36 taxa best described by a two-component model, 33 had a dominant component explaining at least 70% of the distribution, whereas only 3 taxa showed relatively balanced two-component distributions. Thus, although some taxa showed mixed directional tendencies, most taxa were dominated by a primary directional component. As an additional, data-driven check of the original FSI-based classification, we estimated a boundary from the distribution of dominant peak locations (Figure 1—figure supplement 1; Gaussian mixture cutoff ≈ 49.4°). This peak-based classification was identical to the original FSI-based forward/sideways classification (15/35), indicating that the main classification was not dependent solely on FSI.

**Figure 1—figure supplement 1.**
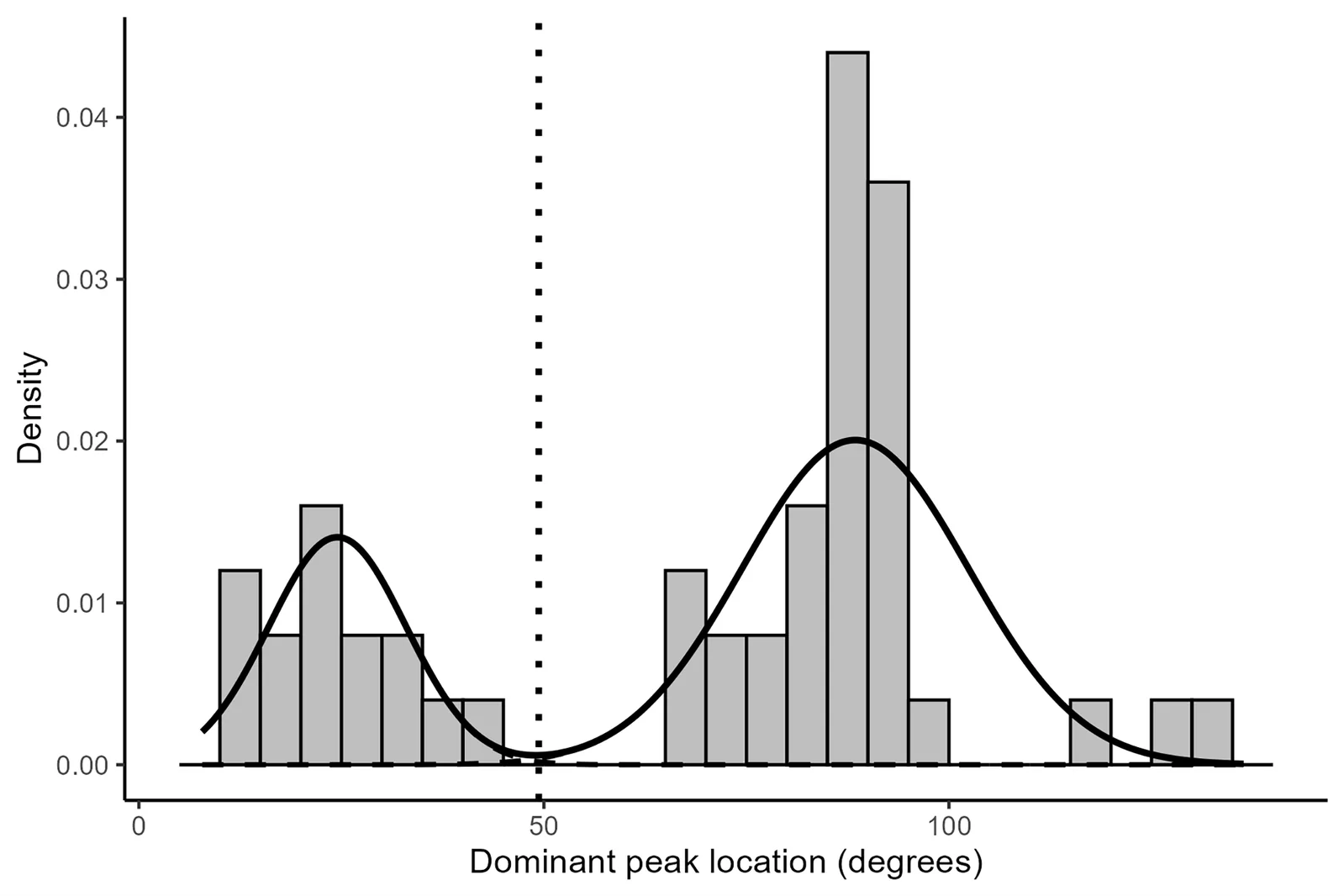
Gaussian mixture fit to dominant peak locations. Dominant peak locations were extracted from mixture models fitted to each taxon’s continuous angle distribution. A two-component Gaussian mixture model fitted to these locations yielded an estimated cutoff at 49.4° (dotted line). Classifying taxa using this data-informed cutoff produced an identical forward/sideways classification to our original approach using Forward–Sideways Index (15 forward, 35 sideways). Figure 1—figure supplement 1—source data 1.Gaussian mixture-model results for continuous bout-angle distributions of the 50 species, including model selection, peak locations, and component weights.

**Figure 1—figure supplement 2.**
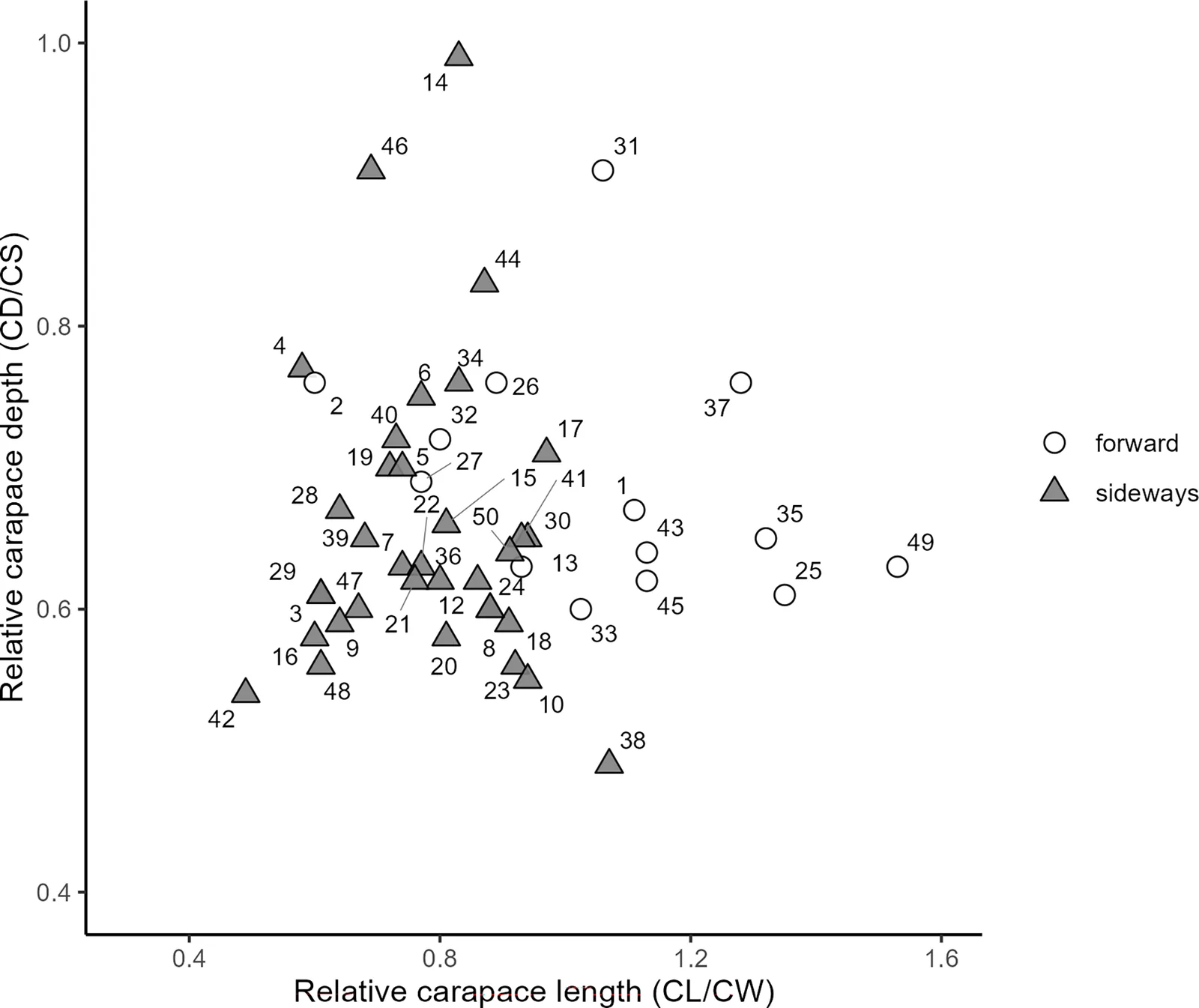
Morphospace of crab locomotor modes based on relative carapace length (CL/CW) and relative carapace depth (CD/CS). Forward- and sideways-moving taxa overlapped broadly in morphospace, although phylogenetically informed ANOVA detected a significant difference in CL/CW but not in CD/CS. CL, carapace length; CW, carapace width; CD, carapace depth; CS, carapace size, defined as the geometric mean of CL, CW, and CD. 1. *Arcania heptacantha;* 2. *Arcotheres sinensis;* 3. *Atergatis floridus;* 4. *Austruca lactea;* 5. *Calappa philargius;* 6. *Cardisoma carnifex;* 7. *Carpilius convexus;* 8. *Chaceon granulatus;* 9. *Charybdis (Charybdis) japonica;* 10. *Chionoecetes opilio;* 11. *Coenobita purpureus;* 12. *Cyclograpsus intermedius;* 13. *Dorippe sinica;* 14. *Dotilla wichmanni;* 15. *Enoplolambrus validus;* 16. *Epixanthus frontalis;* 17. *Erimacrus isenbeckii;* 18. *Eriocheir japonica;* 19. *Eriphia ferox;* 20. *Gaetice depressus;* 21. *Gecarcoidea lalandii;* 22. *Geothelphusa dehaani;* 23. *Grapsus albolineatus;* 24. *Hemigrapsus sanguineus;* 25. *Hyas alutaceus;* 26. *Lauridromia dehaani;* 27. *Lybia tessellate;* 28. *Lydia annulipes;* 29. *Macrophthalmus (Mareotis) japonicus;* 30. *Matuta victor;* 31. *Mictyris brevidactylus;* 32. *Mursia armata;* 33. *Neohymenicus orientalis;* 34. *Ocypode stimpsoni;* 35. *Oregonia gracilis;* 36. *Parasesarma pictum;* 37. *Paromola japonica;* 38. *Percnon planissimum;* 39. *Pilodius areolatus;* 40. *Pilumnus vespertilio;* 41. *Plagusia squamosa;* 42. *Portunus pelagicus;* 43. *Ranina ranina;* 44. *Sayamia germaini;* 45. *Schizophrys aspera;* 46. *Scopimera globosa;* 47. *Scylla serrata;* 48. *Thalamita sima;* 49. *Tiarinia cornigera;* 50. *Xenograpsus testudinatus.*

**Figure 2—figure supplement 1.**
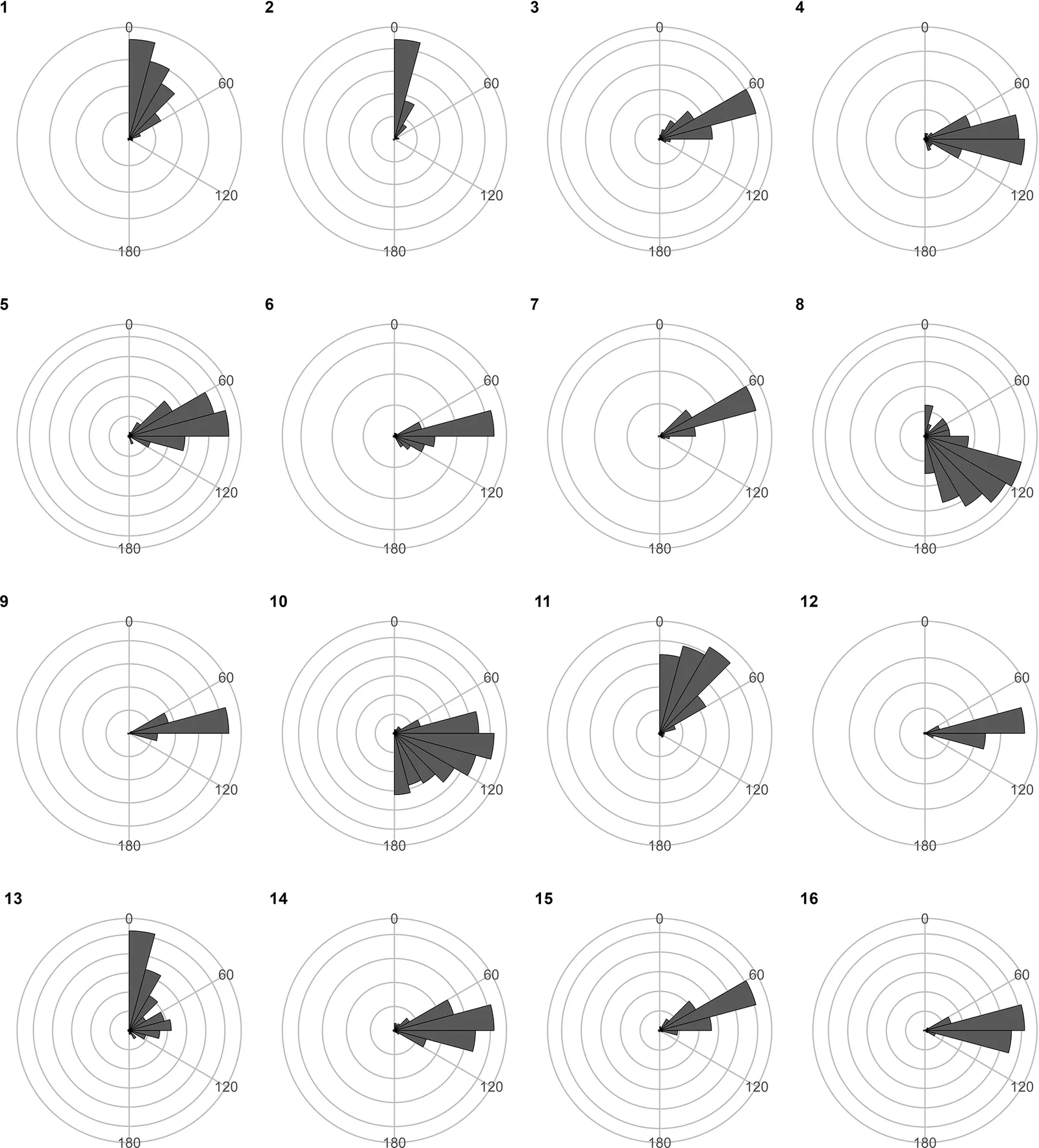
Circular histograms showing movement direction for species 1–16. Numbers in the plot correspond to the species numbers shown in Figure 1—figure supplement 2.

**Figure 2—figure supplement 2.**
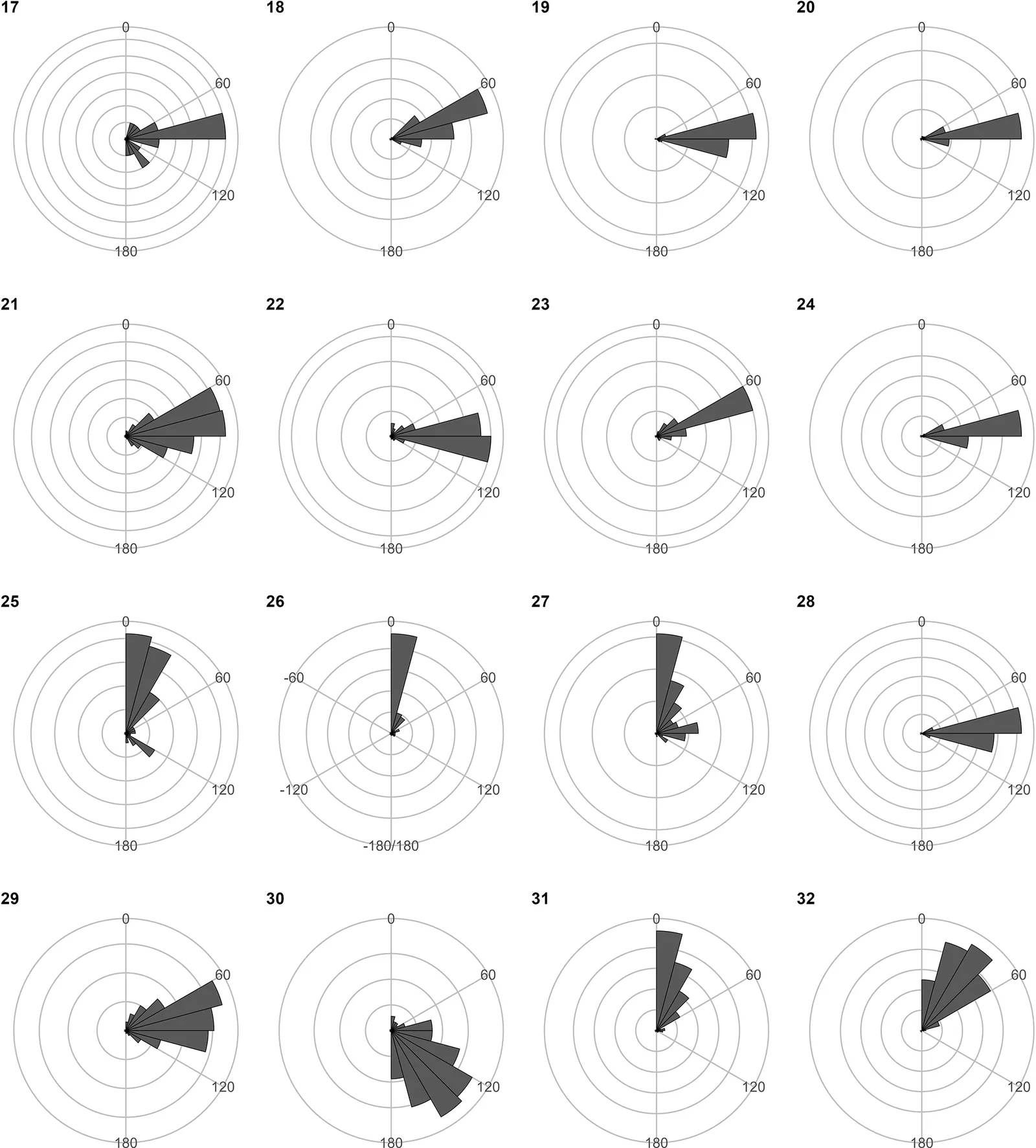
Circular histograms showing movement direction for species 17–32. Numbers in the plot correspond to the species numbers shown in Figure 1—figure supplement 2.

**Figure 2—figure supplement 3.**
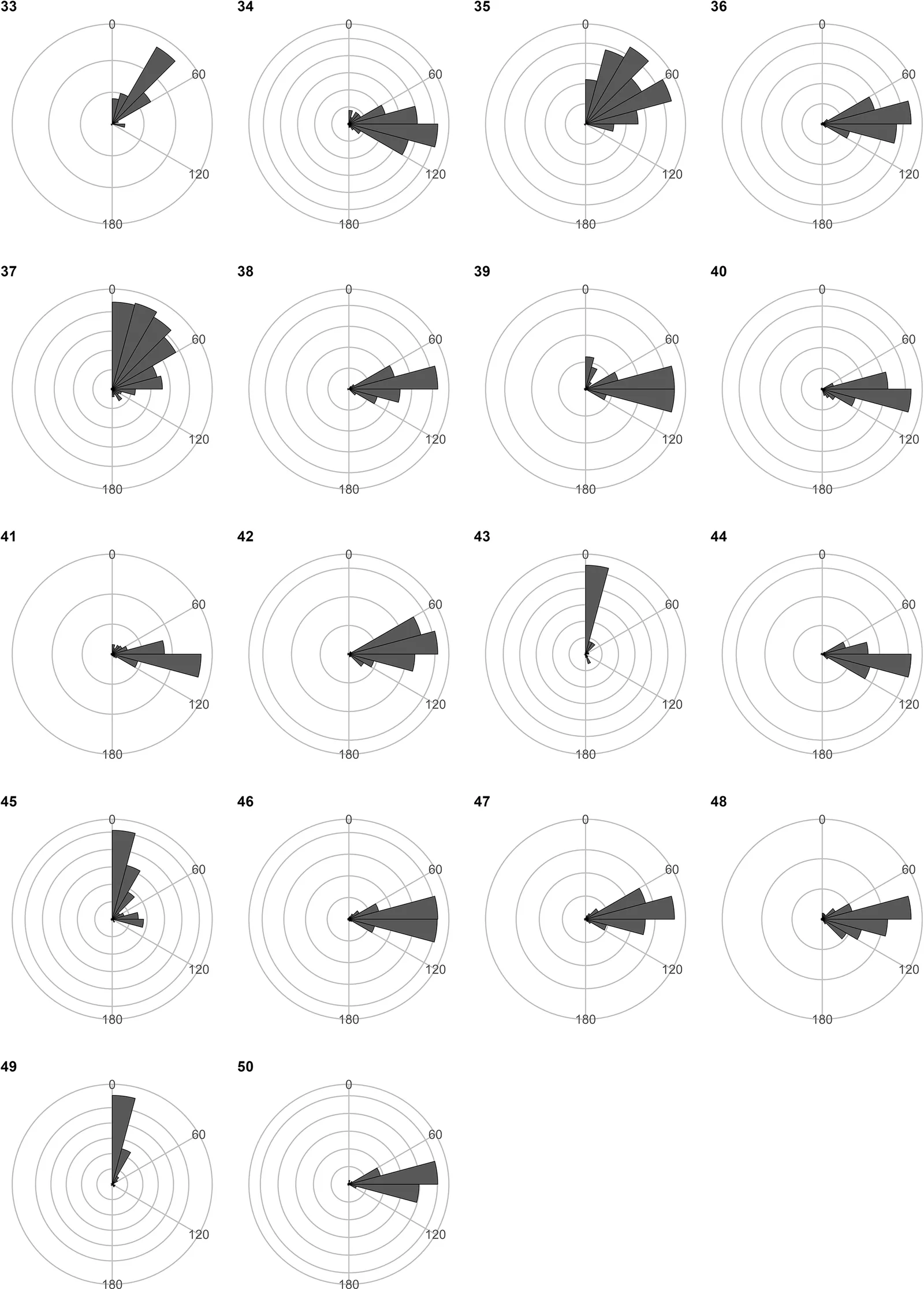
Circular histograms showing movement direction for species 33–50. Numbers in the plot correspond to the species numbers shown in Figure 1—figure supplement 2.

In the morphological analysis (Figure 1—figure supplement 2), relative carapace length (CL/CW, carapace length divided by carapace width) differed significantly between forward- and sideways-moving taxa (phylogenetically informed ANOVA: *F*=26.90, p<0.001), whereas relative carapace depth (CD/CS, carapace depth divided by carapace size) did not differ significantly between the two groups (*F*=1.18, p=0.403). These results suggest that locomotor mode is associated with some aspects of carapace shape, but is not explained by simple carapace flattening alone.

**Figure 3.**
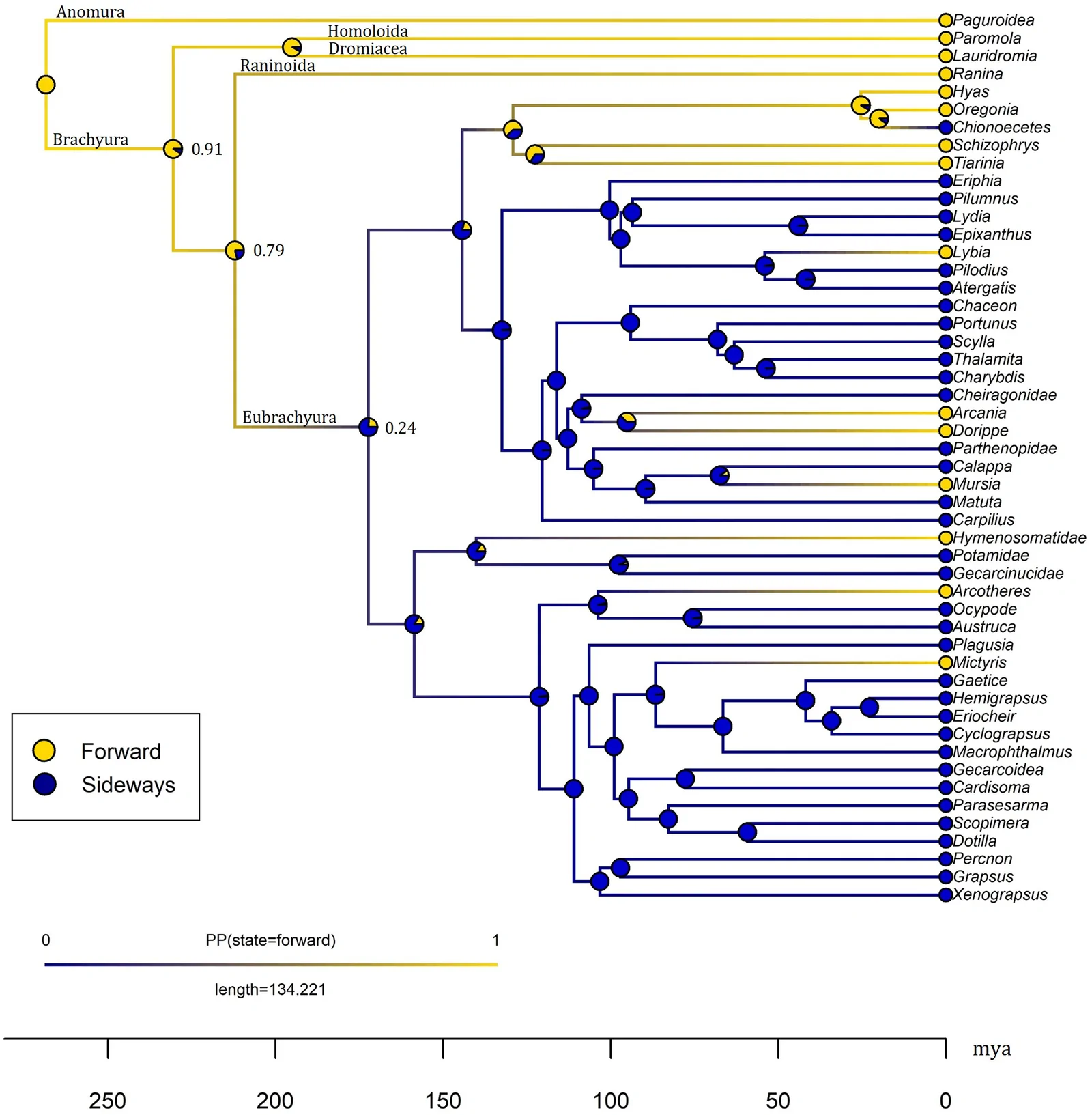
Ancestral state reconstruction of locomotion in crabs under the all-rates-different (ARD) model. Gold circles at the tips indicate forward locomotion, whereas blue circles indicate sideways locomotion. Pie charts at internal nodes and along branches represent the posterior probabilities of each locomotor state, estimated from 500 stochastic character maps. The x-axis shows geological time, scaled in millions of years before present (mya). Figure 3—source data 1.Pruned phylogenetic tree used for the ancestral-state reconstruction in Figure 3, provided in Newick format. Figure 3—source data 2.Continuous FSI values and discrete forward/sideways locomotor states for the 50 terminal taxa used in the ancestral-state reconstruction in Figure 3. Figure 3—source code 1.R code used for stochastic ancestral-state reconstruction of forward and sideways locomotion and generation of Figure 3.

For ancestral-state reconstruction, model comparison using Akaike information criterion (AIC) favored the all-rates-different (ARD) model over the equal-rates (ER) model (ΔAIC = 6.23; AIC weights = 0.957 vs. 0.043). Under the preferred ARD model, the transition rate from sideways to forward locomotion was estimated as 0.0029, slightly higher than the forward-to-sideways rate (0.0026). Ancestral state reconstruction placed the origin of sideways locomotion at the base of Eubrachyura (Figure 3). Stochastic character mapping estimated the posterior probability of forward locomotion as 0.91 for the common ancestor of Brachyura, 0.79 for the common ancestor of Raninoida and Eubrachyura, and 0.24 for the eubrachyuran stem lineage. These results indicate that early-diverging lineages (i.e., Homoloida, Dromiacea, and Raninoida) retained the forward locomotion present in the common ancestor of Brachyura, and that sideways locomotion first appeared at the divergence between Raninoida and Eubrachyura. The ER model yielded a qualitatively similar placement, with only minor differences in the number of inferred transitions (Figure 3—figure supplement 1).

**Figure 3—figure supplement 1.**
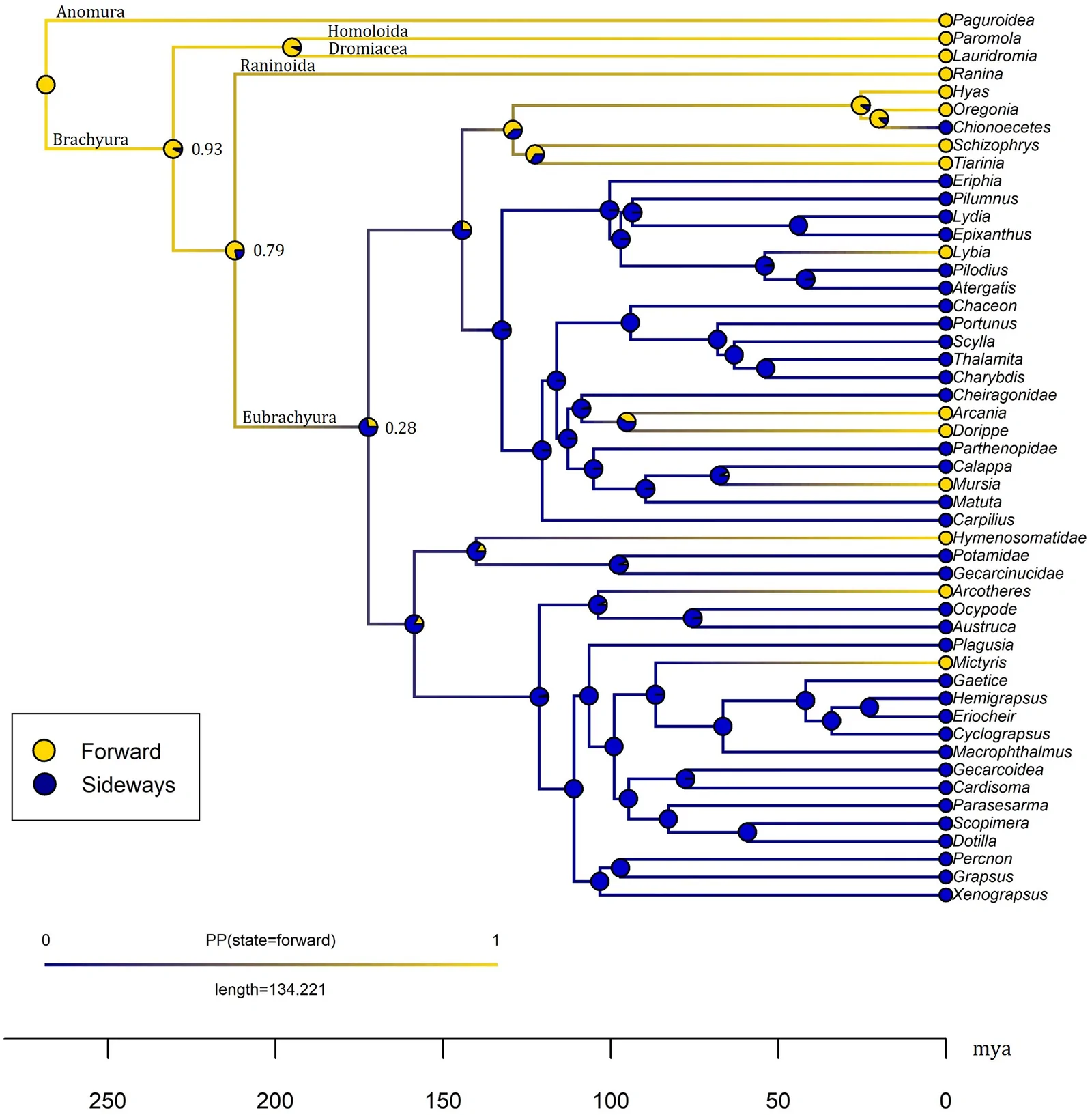
Ancestral state reconstruction of locomotion in crabs under the equal-rates (ER) model. Gold circles at the tips indicate forward locomotion, whereas blue circles indicate sideways locomotion. Pie charts at internal nodes and along branches represent the posterior probabilities of each locomotor state, estimated from 500 stochastic character maps. The x-axis shows geological time, scaled in millions of years before present (mya).

To place these findings in a broader phylogenetic context, we examined locomotor states alongside species richness patterns in early-diverging brachyuran lineages. Cyclodorippoida is identified as the sister group of Eubrachyura, with Raninoida forming the next outgroup (Tsang et al., 2011; Wolfe et al., 2024). Behavioral data from Raninoida indicate that these crabs move forward (Figure 1). In contrast, the locomotor mode of Cyclodorippidae remains unknown because these crabs inhabit deep-sea environments that preclude direct behavioral observations. Based on DecaNet (World List of Decapoda) counts of accepted extant species (DecaNet eds, 2025), Eubrachyura contains approximately 7468 described species, whereas Cyclodorippoida includes about 110 species and Raninoida only ~46 species. This sharp disparity highlights that the clade in which sideways locomotion is fixed—at the base of Eubrachyura or potentially in the common ancestor of Cyclodorippoida and Eubrachyura—is associated with far greater taxonomic diversity than the lineages retaining forward locomotion.

Our results also indicate that the sideways mode was largely retained across major eubrachyuran lineages. However, despite this overall stability, we inferred multiple independent reversions to forward locomotion distributed across the tree, including lineages leading to Majidae (*Hyas*, *Oregonia*, *Chionoecetes*, *Schizophrys*, *Tiarinia*), *Lybia*, *Arcania*, *Dorippe*, *Mursia*, Hymenosomatidae, *Arcotheres*, and *Mictyris*. Interestingly, within Majidae, *Chionoecetes* likely underwent a secondary reversion back to sideways locomotion from a forward-moving ancestor within the group. Stochastic character mapping under the ARD model estimated sideways→forward reversions at a mean of 10.2 (interquartile range, IQR = 9–12) and forward→sideways gains at a mean of 4.1 (IQR = 3–5). Under the ER model, the corresponding estimates were 10.5 (9–12) and 4.6 (3–5), respectively.

These results indicate that the evolution of sideways locomotion is rare but, once established, tends to be stably retained. Reversions to forward locomotion (and subsequent reversions back to sideways locomotion) occur under particular ecological specializations, producing a complex evolutionary pattern across true crabs.

## Discussion

Our ever-biggest behavioral dataset on crab locomotion reveals that sideways locomotion originated only once from the forward locomotion ancestor, rather than through multiple independent origins (Figure 3). In other words, the widespread sideways locomotion across true crabs is highly conserved after being inherited from the common ancestor at the base of Eubrachyura. This suggests that modern sideways-moving crabs likely share the same anatomical, neurological, and developmental mechanisms, such as the reduction of motor neurons that control muscles of proximal legs (Vidal-Gadea and Belanger, 2013). The single transition event of sideways locomotion contrasts with carcinization, which has occurred repeatedly across decapods (Tsang et al., 2011; Keiler et al., 2017; Tan et al., 2018; Wolfe et al., 2021). Carcinization has produced crab-like morphologies in several lineages of Anomura, including porcelain crabs (Porcellanidae), king crabs (Lithodidae), and the coconut crab *Birgus latro*. However, our behavioral observations indicate that porcelain crabs move predominantly backward rather than sideways, and king crabs and the coconut crab move predominantly forward (unpublished data). These examples demonstrate that even when crab-like body forms evolve, the characteristic locomotor mode (i.e., moving sideways) does not necessarily accompany them. This highlights a distinction between morphological convergence and behavioral innovation: while body forms may converge multiple times, fundamental behavioral transitions can be rare.

The species richness data show that Eubrachyura is far more diverse than its nearest outgroups, and the origin of sideways locomotion is inferred to lie around the base of the Eubrachyura. These results are consistent with the idea that this unique locomotor mode acted as a key innovation that contributed to the evolutionary diversification of Eubrachyura. Note, however, that a key innovation is not the only process driving adaptive radiation, and the innovation may not always result in radiation (Fürsich and Jablonski, 1984; Miller et al., 2023). External factors, such as ecological opportunity provided by mass extinction, are also critical for evolutionary diversification (Stroud and Losos, 2016). Based on the divergence times reported by Wolfe et al., 2024, the origin of sideways locomotion falls around 200 million years ago (earliest Jurassic, immediately post–Triassic–Jurassic extinction), a recovery interval marked by Pangaean rifting, expansion of shallow-marine habitats, and the early Mesozoic Marine Revolution—conditions that typically increase ecological opportunity (Buatois et al., 2016; Schoepfer et al., 2022). Disentangling the relative roles of intrinsic innovation and extrinsic environmental change will require trait-dependent diversification analyses (Maddison et al., 2007), fossil-informed timelines, and performance tests that link sideways movement to adaptive advantages.

Sideways locomotion may confer several adaptive advantages. One likely advantage is the ability to move rapidly at similar speeds in both lateral directions (Vidal-Gadea et al., 2008; Wolfe et al., 2021), which is also supported by an experiment using crab-like robots (Chen et al., 2022). Having multiple locomotor directions is highly advantageous for escaping from predators, not only by making the escape direction unpredictable but also by providing multiple optimal escape routes (Domenici et al., 2011; Kawabata et al., 2023). Other possible advantages may include movement through confined spaces and visual-field sampling during locomotion, although these possibilities remain untested and will require future biomechanical and sensory studies. An alternative explanation is that sideways locomotion is a secondary phenomenon resulting from a flattened body structure that limits the distance between the legs and thereby makes forward movement difficult. However, our additional morphological analysis showed that relative carapace depth (CD/CS) did not differ significantly between forward- and sideways-moving taxa, whereas relative carapace length (CL/CW) differed significantly. Therefore, sideways locomotion cannot be explained solely by carapace flattening, and the morphological correlates of sideways locomotion are more complex.

Despite these potential adaptive advantages, sideways locomotion appears to have evolved rarely across the animal kingdom; it has occurred only in true crabs (Figure 3), and potentially also in crab spiders (Wilcox, 2017) and leafhopper nymphs (Chasen et al., 2014). One possible reason is that this locomotor mode fundamentally changes the behavioral axis, potentially affecting a wide range of behaviors such as predator avoidance, shelter use, burrowing, mating, and foraging (Atkinson and Eastman, 2015; Crane, 2015; Asakura, 2016; Takeshita and Nishiumi, 2022). In this sense, the origin of sideways locomotion may represent a rare and major behavioral transition rather than a simple change in movement direction alone.

Nevertheless, sideways locomotion does not appear to be universally favored across all ecological contexts. After the transition to sideways locomotion, crabs have experienced at least six independent reversions to forward locomotion (Figure 3). These reversions are particularly associated with major changes in life history traits. For example, soldier crabs (*Mictyris*) predominantly use forward walking (Figure 2—figure supplement 2, Figure 1—source data 1) in a way biomechanically similar to other forward-walking animals rather than sideways-walking crabs (Sleinis and Silvey, 1980). Soldier crabs are unique for their gregarious nature and coordinated collective movements (Murakami et al., 2014), which may have brought them back to forward locomotion. Similarly, majoid crabs (e.g., *Oregonia, Tirarinia*) camouflage themselves with seaweed (Sato and Wada, 2000; Hultgren and Stachowicz, 2009), and pea crabs (e.g., *Arcotheres*) live hidden inside bivalves and other invertebrates, relying on their hosts for protection (de Gier and Becker, 2020). Given that the major benefit of sideways locomotion is rapid escape from predators (Vidal-Gadea et al., 2008; Wolfe et al., 2021), these examples with alternative strategies of predator avoidance may no longer need sideways locomotion, resulting in secondary losses. These exceptional forward-moving species imply that it is costly to maintain sideways locomotion in crabs, and that they retain evolutionary flexibility to lose this locomotor mode under certain ecological pressures.

Modes of locomotion—such as walking, swimming, and flying—fundamentally shape how animals interact with the environment, affecting behaviors related to foraging, predator avoidance, and reproduction (Alexander, 2003; Domenici, 2010). Sideways locomotion in true crabs is also a critical change in the mode of locomotion, whose evolutionary history is characterized by rarity, stability, and occasional reversions. Our case study illustrates how major innovations can open new adaptive opportunities, yet remain constrained by both phylogenetic history and ecological context. By integrating direct behavioral observations with a robust phylogenetic framework, this study expands our understanding of how animal locomotor modes diversify and persist through evolutionary time.

## Materials and methods

### Experimental procedures

We obtained live crabs from multiple sources, including intertidal and subtidal field collections, public aquaria, and local fish markets. Animals were kept only as long as required to record locomotion and were returned to their habitat or handled according to institutional animal care guidelines. Animal care and experimental procedures were approved by the Animal Care and Use Committee of the Faculty of Fisheries, Nagasaki University (Permit No. NF-0060), in accordance with the Guidelines for Animal Experimentation of the Faculty of Fisheries and the Regulations of the Animal Care and Use Committee of Nagasaki University.

**Figure 4.**
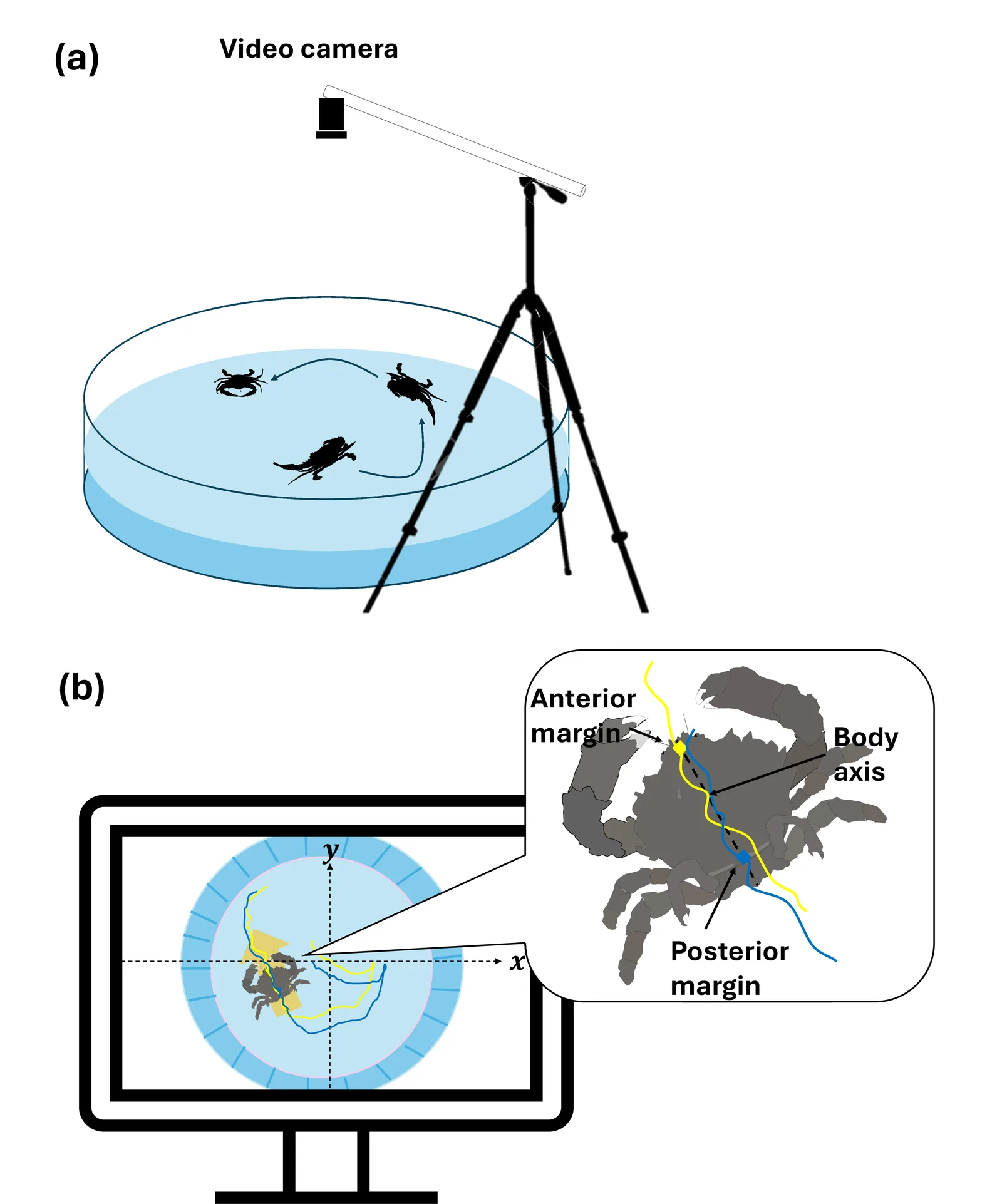
Video acquisition and analysis workflow. (**a**) Experimental setup used to record each crab’s behavior. (**b**) Extraction of two-dimensional position coordinates from video frames.

Locomotion was recorded in plastic circular arenas (diameter 80–140 cm) whose medium matched each species’ native environment (dry, seawater, freshwater, or brackish, with or without bare sand) (Figure 4a). Individuals were acclimated for 5 min in a bucket and then for 1 min inside a transparent cylinder placed at the arena center to minimize startle responses. After removing the cylinder, each trial was filmed for 10 min using a standard video camera (DSC-RX0, Sony Corporation, Tokyo, Japan) at 30 frames s⁻¹. The locomotion data were obtained from one representative individual per species due to logistical constraints and the low expected within-species variation. Our preliminary observations on several species for which many individuals were available suggest that locomotor direction is a species-level trait that is typically conserved. Nevertheless, this design does not capture possible ontogenetic, size-dependent, or allometric variation within species. Accordingly, our conclusions are intended to identify broad interspecific patterns of locomotor direction rather than the full range of within-species variation.

### Video analysis

Obtained videos were converted and downsampled to 5 frames s⁻¹ for analysis using XMedia Recode 3.5 (https://www.xmedia-recode.de/). For each frame, two landmarks along the longitudinal body axis (anterior and posterior carapace margins) were digitized using Kinovea 0.8.27 (https://www.kinovea.org/) to estimate the instantaneous body axis and the centroidal position of the animal (Figure 4b).

**Figure 5.**
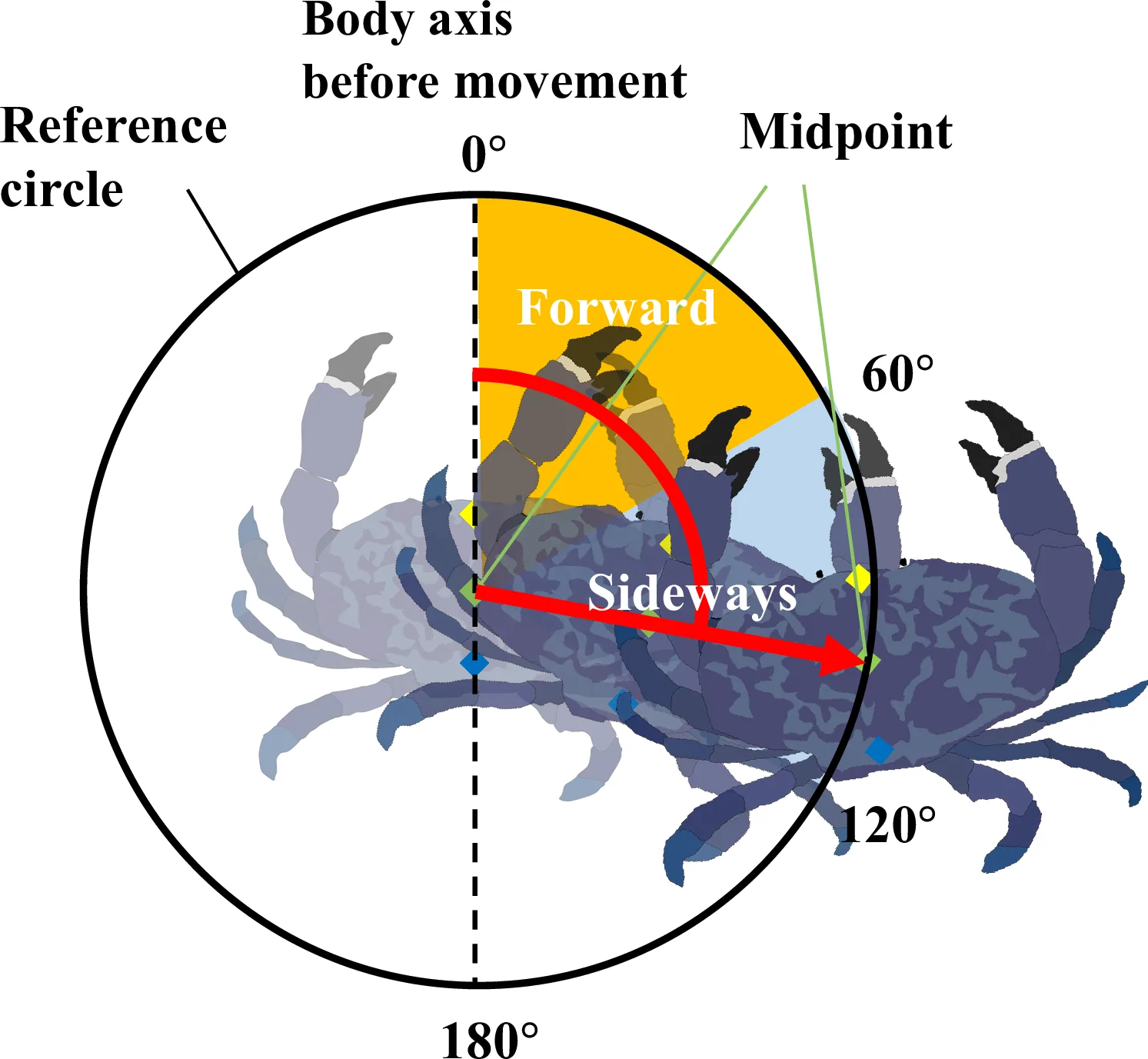
Method for determining movement directions of each crab. For each movement bout, movement direction was defined as the angle between the previous body axis (from tail to head) and the displacement vector of the body’s center (referred to as the midpoint). Displacement was measured when the midpoint reached the reference circle. These values across all movement bouts were then used to calculate the Forward–Sideways Index (FSI).

To standardize the evaluation of movement directions, we used a reference circle centered on the animal’s starting position (Figure 5). Each displacement bout was defined as the movement from the starting point to the point where the trajectory of the body center crossed the circle, with the next bout beginning once the reference circle was crossed. For each displacement bout, we computed the angle between the pre-movement body axis and the line to the crossed point as the movement direction (Figure 5). Movements occurring to the left of the body axis were mirrored to the right before classification. Bouts were classified as forward (0–60° relative to the body axis), sideways (60–120°), or backward (>120°). This classification reflects three equal directional sectors in the full 360° movement space (forward/sideways/backward; 120° each), which provides a consistent reference under a null expectation of uniform movement directions. Backward movements were rare and therefore excluded from the analyses (Figure 2—figure supplements 1–3). We calculated behavior using the FSI: FSI = (F − S)/(F+S), where F denotes the number of forward bouts and S denotes the number of sideways bouts. FSI values range from −1 (completely sideways) to +1 (completely forward). Species with FSI <0 were classified as sideways movers, and those with FSI >0 as forward movers.

The radius of the reference circle was determined systematically for each species (Figure 1—source data 1). For each species, we tested circle radii from 2 mm to 200 mm in 2 mm increments and computed the FSI at each radius. When the circle is too small, digitizing noise and body wobble near the start point tend to drive FSI toward 0. When the circle is too large, animals may turn within the circle before crossing, making the estimate unstable and unreliable. To capture the biologically meaningful motions, we selected the smallest radius at which the FSI showed a clear local maximum or minimum away from zero and then remained approximately stable as the radius increased further.

### Ancestral state reconstruction

We extracted and pruned a recently published crab phylogeny (Wolfe et al., 2024), which was based on sequences of 10 genes (2 mitochondrial ribosomal RNA coding genes, 2 nuclear rRNA genes, and 6 nuclear protein-coding genes) and included 344 species across most major brachyuran lineages. Because our behavioral dataset did not always perfectly match the species included in Wolfe et al., 2024, we reduced this tree to 44 genera, 5 families, and 1 superfamily, allowing closely related taxa to represent the observed species when the same species were unavailable. This approach enabled us to retain the terminals present in our dataset while preserving the placements of major clades relevant to this study (e.g., Eubrachyura, Raninoida).

All terminals (44 genera, 5 families, and 1 superfamily) were coded as discrete states of either forward or sideways, based on the observed FSI values (positive: forward, negative: sideways). The tree was rooted with a hermit crab terminal (Anomura), for which we assigned the observed locomotor mode of *Coenobita purpureus* from our behavioral data. The node representing the common ancestor of Brachyura and Anomura (Meiura, starting/root node) was fixed to forward as a root prior. This assumption is based on the fact that further outer groups (e.g., crayfish, Astacidea) exhibit forward locomotion (Vidal-Gadea et al., 2008). Ancestral states were estimated on the pruned tree using maximum likelihood under ER and ARD models, with model fit compared by AIC. To summarize node-wise uncertainty and transition counts, we performed stochastic character mapping (500 replicates) and reported posterior probabilities at key nodes and the posterior distributions of transitions and reversals. All analyses were conducted in R version 4.3.2 (R Development Core Team, 2023) using the packages *ape*, *phytools*, and *geiger*.

### Morphological analysis

To examine the relationship between body shape and locomotor mode, we used two simple carapace shape indices: relative carapace length (CL/CW, carapace length divided by carapace width) and relative carapace depth (CD/CS, carapace depth divided by carapace size), where carapace size (CS) was defined as the geometric mean of CL, CW, and CD, where CL is carapace length, CW is carapace width, and CD is carapace depth. We used phylogenetically informed ANOVA to test whether these indices differed between forward- and sideways-moving taxa.

## Data Availability

Figure 1—source data 1, Figure 1—figure supplement 1—source data 1, Figure 2—source data 1–5, Figure 3—source data 1 and 2 contain the numerical data used to generate the figures. Source code files used for the analyses are provided with the article. The following previously published dataset was used: WolfeJ BallouL LuqueJ Watson-ZinkV AhyongS Barido-SottaniJ ChanT ChuK CrandallK DanielsS FelderD ManckeH MartinJ NgP Ortega-HernándezJ Palacios TheilE PentcheffD RoblesR ThomaB TsangL WetzerR WindsorA Bracken-GrissomH 2023Convergent adaptation of true crabs (Decapoda: Brachyura) to a gradient of terrestrial environmentsDryad Digital Repository10.5061/dryad.tmpg4f52zPMC1128236637941464

## Additional files

MDAR checklist
