# Supplementary material for: Evolution of sideways locomotion in crabs
Source: eLife. 2026 Aug 27;15:RP110015. doi: 10.7554/eLife.110015 (PMC13521562; doi:10.7554/eLife.110015)
Supplement: Peer review history

# Editor report: eLife Assessment

**DOI:** 10.7554/eLife.110015.3.sa0

**Authors:** Yuuki Y Watanabe

**Affiliations:** Graduate University for Advanced Studies, SOKENDAI Japan

**Keywords:** Convincing, Valuable

This **valuable** study presents a comparative dataset on crab locomotion to investigate the evolution of sideways walking. The evidence supporting the authors' claims is **convincing**. This work will be of broad interest to researchers in animal locomotion and evolutionary biology.

# Referee report: Reviewer #1 (Public review)

**DOI:** 10.7554/eLife.110015.3.sa1

Summary:

This is an interesting and well-written manuscript in which the authors set out to answer a simple, longstanding question with a modern comparative approach. Namely where in crab evolution did sideways walking arise, how often has it been lost or regained, and is its evolution plausibly associated with the ecological and taxonomic success of true crabs. To address these questions the authors recorded locomotion from 50 live species, quantified the predominant direction of locomotion, and mapped these behavioral states onto a recent crab phylogeny to reconstruct the likely evolutionary history of sideways walking. The revised manuscript also includes analyses of the underlying movement-angle distributions and tests whether the evolutionary conclusions depend on the original behavioral classification scheme.

Strengths:

The strongest part of the study remains the dataset itself. Comparable behavioral measurements across dozens of crab species are rare, and obtaining and recording live representatives from this range of taxa required substantial field, aquarium, and husbandry effort. The overall pattern that emerges, in which most true crabs are strongly biased toward sideways locomotion while several specialized lineages move predominantly forward, is interesting and likely to be useful to researchers studying animal locomotion, functional morphology, and behavioral evolution.

The revised analyses substantially strengthened the manuscript. In the original version, I was concerned that the main behavioral classification depended too strongly on first assigning individual movements to forward or sideways bins using a fixed angular boundary. The authors have now analyzed the underlying continuous movement-angle distributions and have shown that, although mixed directional tendencies are present in some species, most taxa have a dominant directional preference. They also derived a separate, data-informed boundary from the distribution of dominant movement directions. I favor this alternative approach and it produces the same classification of species as the original index-based method, providing useful evidence that the main evolutionary reconstruction is not simply an artifact of the original 60{degree sign} cutoff.

The authors also responded appropriately to the limitation that locomotion was measured from one individual per species. This sampling design cannot establish the full extent of within-species, ontogenetic, or size-dependent variation, but the revised manuscript now states this limitation clearly and restricts its conclusions to broad interspecific patterns in predominant locomotor direction. This is a more appropriate interpretation of the available sampling.

The phylogenetic analysis provides a reasonable framework for addressing the main evolutionary question. Taken together, the behavioral and phylogenetic results support the conclusion that sideways locomotion likely arose once within the lineage leading to true crabs and was followed by multiple reversions toward predominantly forward locomotion in specialized groups. The manuscript therefore makes a convincing case that sideways walking is not simply an inevitable consequence of possessing a crab-like body plan.

Weaknesses:

My main remaining reservation concerns the interpretation of forward and sideways locomotion as two discrete biological modes. The revised analyses convincingly show that species can be classified according to their predominant direction of locomotion and that this classification is robust to alternative analytical approaches. However, this does not necessarily demonstrate that forward and sideways locomotion represent two intrinsically discrete or mutually exclusive behavioral modes. Indeed, the new analyses show that many taxa are better described by two-component movement-angle distributions, even though most of these have one dominant component. This is consistent with strong directional preferences, but it also indicates that mixed movement strategies are common. The supplementary circular distributions similarly show considerable variation in the shape and breadth of directional preferences among taxa. Having said that, I do not think this substantially weakens the central evolutionary conclusion. The phylogenetic analysis requires a defensible classification of predominant locomotor direction, and the revised analyses now provide one. The evolutionary story remains interesting whether the underlying behavioral variation consists of two sharply discrete modes or a broader continuum of directional strategies with strong clustering toward forward and sideways movement.

A second limitation is that the proposed relationship between sideways locomotion and diversification remains necessarily correlational. The revised manuscript handles this more cautiously than the original version and now frames sideways locomotion as a plausible key innovation whose emergence is associated with the exceptional diversity of true crabs, rather than as a demonstrated causal driver of diversification. This distinction is important because differences in species richness among lineages can also reflect ecological opportunity, extinction history, and other lineage-specific factors. The revised framing is therefore better aligned with the strength of the evidence.

Final assessment: Overall, this is a valuable comparative study with an unusually broad behavioral dataset. The revisions have addressed the principal methodological concerns raised in the original review, particularly by analyzing continuous movement directions and demonstrating that the main phylogenetic classification is robust to an alternative, data-informed approach. The evidence now convincingly supports the central conclusion concerning the evolutionary origin and repeated reversal of predominant locomotor direction in crabs, although the stronger interpretation that forward and sideways locomotion represent two strictly discrete biological modes remains less certain.

# Referee report: Reviewer #2 (Public review)

**DOI:** 10.7554/eLife.110015.3.sa2

Summary:

The current work investigates the evolution of sideward locomotion in Brachyura in light of a single evolutionary origin. To this end, the authors first analysed the mode of locomotion in 50 crab species and observed mutually exclusive presence of sideways vs. forward movement. The phylogenetic analysis confirmed that there is indeed a single evolutionary origin for sideways movement, which was sometimes followed by several reversions to forward locomotion. This way, authors demonstrate how locomotor movement modes shape evolutionary diversification in animals by showing that species richness is much higher in side-ways-moving crabs than in the nearest groups. This is an interesting work that integrates behavioural analysis and phylogenetic relations, capitalising largely on crabs.

Original questions/suggestions:

Firstly, I think the paper spends too much time on a straightforward analysis of the mode of locomotion. I was also wondering whether the phylogenetic analysis could be simply achieved by maximising an objective function in which the modes of movement are inversely coded for two putative groups, with all values calculated at all possible nodes.

Unfortunately, I find that the authors did not sufficiently discuss differences in the ecological niches of species with forward vs. sideways locomotion modes (including challenges of locomotion and substrate).

Likewise, what are the anatomic correlates of forward vs. sideways locomotion? For instance, how are the advantages assumed for sideways movement associated with a flattened body? Is it possible that the mode of motion is secondary to flattened/narrow body structure, which basically limits the distance between legs and thus makes the forward movement difficult - under this logic, the mode of movement would be a secondary phenomenon to body shape traits. How can one differentiate between this alternative and the one that puts the mode of movement in the centre of the story? On a related note, how do different modes of movement relate to the ability to fit into tight spaces - how does it relate to differences in leg joints?

Is it possible that the sideways movement maximises the scanned visual field per unit time/displacement, which may be beneficial for mostly forward-moving predators?

Briefly, although I find the study interesting, the presented complexity may not be necessary given the endpoints; it can be achieved much more simply. Furthermore, the degree to which the conceptual analysis of different modes of locomotion was exercised was limited. The general approach may serve as a good model for the evolutionary analysis of other traits. The demonstration of traceability of the relations in question is a major contribution of the work.

Comment on revised version:

I am not fully convinced by the authors' handling of the complexity of the paper, but this seems like a moot point.

# Author comment: Author response

**DOI:** 10.7554/eLife.110015.3.sa3

**Authors:** Junya Taniguchi, Tsubasa Inoue, Kano Kohara, Jung-Fu Huang, Atsushi Hirai, Nobuaki Mizumoto, Fumio Takeshita, Yuuki Kawabata

**Affiliations:** Graduate School of Integrated Science and Technology, Nagasaki University Nagasaki Japan; Graduate School of Integrated Science and Technology, Nagasaki University Nagasaki Japan; Graduate School of Integrated Science and Technology, Nagasaki University Nagasaki Japan; National Kaohsiung University of Science and Technology Kaohsiung Taiwan; Susami Crustacean Aquarium Wakayama Japan; Okinawa Institute of Science and Technology Graduate University Auburn United States; Kitakyushu Museum of Natural History and Human History Fukuoka Japan; Nagasaki University Nagasaki Japan

The following is the authors’ response to the original reviews.

**Public Reviews:**

**Reviewer #1 (Public review):**

Summary:

This is an interesting and well-written manuscript in which the authors set out to answer a simple, old question with a modern toolkit: where in crab evolution did sideways walking arise, how often has it been lost or regained, and is it plausibly linked to the ecological and taxonomic success of true crabs. To do this, they record locomotion from 50 live species, convert each species' movements into a quantitative index that compares forward versus sideways bouts, and then map the resulting states onto a recent crab phylogeny to infer the most likely evolutionary history of locomotor direction.

We thank the reviewer for this positive summary of the study and for recognizing the value of our comparative behavioral dataset and phylogenetic approach.

Strengths:

The strongest part of the study is the dataset itself. Comparable behavioral measurements across dozens of crab species are rare. The authors have done the field and husbandry work needed to make this possible. The overall pattern they recover, that most true crabs are strongly biased toward sideways movement (while a smaller set of lineages move predominantly forward), is interesting and likely to be useful to others. The phylogenetic mapping is also a reasonable way to address the "how many times" question (although this is peripheral to my expertise). The manuscript makes a convincing case that sideways locomotion is not simply a trivial byproduct of a crab-like body plan.

We appreciate the reviewer’s recognition of the dataset and the overall value of the study. We have revised the manuscript to make the conclusions more robust and better aligned with the strength of the evidence.

(1) Where I am less convinced is in how strongly the authors describe the discreteness of the behavioral categories and the absence of intermediates. The manuscript states that the Forward-Sideways Index shows a clear separation between two locomotor types with little evidence for intermediates, and it cites a statistical test rejecting a single peak in the distribution. However, the histogram in Figure 3 appears structured within each labeled category, with subclusters inside both the forward and sideways groups rather than a single tight peak per group. This matters because the index is built by first placing each movement bout into "forward" versus "sideways" bins using a fixed angle boundary and then collapsing the result into a single ratio. That approach is simple and transparent enough, but it can also hide mixed strategies. For example, a species that produces substantial amounts of both forward and sideways walking can still end up with a strongly positive or negative index, and therefore be classified as a pure "type," even though the underlying behavior is mixed. In that context, rejecting a single peak in the across-species distribution does not, by itself, justify the stronger claim that intermediates are rare or absent.

Related to this, a key methodological choice is the use of 60 degrees as the cutoff between forward and sideways bouts. This boundary may be reasonable as a convention, but the paper does not explain why it is the right place to draw the line, and there is a plausible biological concern that a fixed angular cutoff does not mean the same thing across taxa.

Crabs vary in body shape and in how the legs are arranged around the body. In my own comparative work, for example, some species show an elliptical stance pattern elongated along the preferred direction of travel, while others show a more circular leg arrangement, and the latter can express more mixed forward and sideways behavior. When limb arrangement and body geometry differ across species, the same measured angle can correspond to different underlying mechanics and different functional "degree of sidewaysness." The practical implication is that the reported binary separation may partly reflect the imposed classification rule, rather than a sharp biological divide.

We thank the reviewer for this important point. We agree that the across-species distribution of FSI values alone does not justify a strong statement that intermediate or mixed locomotor tendencies are absent. We also agree that reducing continuous bout-angle distributions to a single index could potentially obscure mixed directional strategies. We have therefore revised the manuscript to avoid implying a strict absence of intermediates and have added an additional analysis of the underlying continuous angle distributions (Abstract, lines 27-29; Results, lines 191-208; Table S2).

Specifically, we fitted one- and two-component mixture models to the continuous bout-angle distributions of each taxon and examined the supported number of components, peak locations, and mixture weights (Results, lines 196-204; Table S2). This analysis showed that 14 taxa were best described by a one-component model, whereas 36 taxa were best described by a two-component model. Importantly, among the 36 taxa best described by a two-component model, 33 had a dominant component explaining at least 70% of the distribution, whereas only three taxa showed relatively balanced two-component distributions. Thus, although some taxa do show mixed directional tendencies, most taxa are dominated by a primary directional component rather than showing an even mixture of forward and sideways locomotion.

We also clarified the rationale for the 60° threshold used in the FSI calculation (Methods, lines 125-130). This threshold was not intended to represent a taxon-specific biological boundary between forward and sideways locomotion. Rather, it was used to divide the 360° space into three equal directional sectors: forward, sideways, and backward. This equal partitioning provides a consistent reference under a null expectation of uniformly distributed movement directions.

To further assess whether our classification depended on the original FSI-based classification, we performed an additional data-driven check based on continuous bout-angle distributions (Results, lines 204-208; Fig. S2). We extracted the dominant peak location from each taxon’s continuous bout-angle distribution (Table S2) and estimated a boundary from the distribution of these dominant peak locations using a Gaussian mixture model. This yielded a data-informed cutoff of approximately 49.4°. The resulting peak-based classification was identical to the original FSI-based classification, with 15 forward-moving and 35 sideways-moving taxa. Thus, no taxon changed category under this independent classification approach.

(2) Another limitation that affects interpretation is the decision to use one individual per species. I understand the logistics, and for some questions, a single representative individual can be a reasonable first pass. But it is not strong support for negative claims about intermediates, especially in a group where individuals can change substantially with growth and allometry. Crabs can grow dramatically, often with pronounced allometric shifts in limb proportions that can alter the center of mass location. Size alone can alter the kinematics and choice of locomotor behaviors in crustaceans. In species where appendage proportions change with size, or where certain legs become disproportionately large (or calcified), it is plausible that locomotor direction and the distribution of movement angles shift across ontogeny. That makes it hard to treat a single individual as a complete description of a species-level strategy, particularly for species that fall closer to the boundary between categories.

We thank the reviewer for raising this important limitation. We agree that using one representative individual per species cannot capture the full range of within-species variation, including ontogenetic, size-dependent, or allometric changes in locomotor behavior. We also agree that this limitation is particularly relevant to strong claims about the absence of intermediates.

As described in our response to Comment #1, we have therefore toned down statements implying a strict absence of intermediates and added analyses of the underlying continuous angle distributions (Abstract, lines 27-29; Results, lines 191-208; Table S2). These additional analyses showed that some taxa do exhibit mixed directional tendencies, although most taxa were dominated by a primary directional component.

We have also revised the manuscript to clarify the scope of our conclusions. Specifically, we now state that our single-individual sampling design does not capture possible ontogenetic, size-dependent, or allometric variation within species (Methods, lines 105-107). We also clarify that our conclusions are intended to identify broad interspecific patterns in the predominant direction of locomotion across major brachyuran lineages, rather than to describe the full range of locomotor variation within each species (Methods, lines 107-108). Thus, we no longer treat a single individual as providing a complete description of species-level behavioral variation, but instead use it as a standardized representative observation for broad comparative and phylogenetic analyses.

In sum, this is a valuable and useful behavioral comparative study with a dataset that many in the field will appreciate. The main conclusions about the likely evolutionary placement of sideways walking are plausible, but several of the stronger claims about discrete locomotor types, the absence of intermediates, and the relationship to diversification would be more convincing if the analysis were less dependent on a fixed angular cutoff and on single individuals per species, or if the manuscript framed those points more cautiously so the conclusions track the strength of the evidence.

We thank the reviewer for this constructive summary and for recognizing the value of our behavioral comparative dataset. We have addressed these concerns in detail in our responses above and revised the manuscript to make the main claims better aligned with the strength of the evidence.

**Reviewer #2 (Public review):**

Summary:

The current work investigates the evolution of sideward locomotion in Brachyura in light of a single evolutionary origin. To this end, the authors first analysed the mode of locomotion in 50 crab species and observed mutually exclusive presence of sideways vs. forward movement. The phylogenetic analysis confirmed that there is indeed a single evolutionary origin for sideways movement, which was sometimes followed by several reversions to forward locomotion. This way, authors demonstrate how locomotor movement modes shape evolutionary diversification in animals by showing that species richness is much higher in side-ways-moving crabs than in the nearest groups. This is an interesting work that integrates behavioural analysis and phylogenetic relations, capitalising largely on crabs. I have a few suggestions and questions.

We thank the reviewer for the positive assessment of the study and for recognizing the value of integrating behavioral analysis with phylogenetic relationships. We address the specific suggestions and questions below.

(1) Firstly, I think the paper spends too much time on a straightforward analysis of the mode of locomotion.

We agree that the final classification of taxa into predominantly forward- and sideways-moving groups is conceptually simple. However, because our study compares locomotor behavior across a broad range of crab taxa and then uses these behavioral data for phylogenetic reconstruction, we considered it important to describe the behavioral quantification in a transparent and reproducible way. The purpose of this section is therefore not to make a simple endpoint unnecessarily complex, but to show how discrete locomotor states were derived from raw trajectory data using a standardized procedure. For this reason, we retained the current analytical description.

(2) I was also wondering whether the phylogenetic analysis could be simply achieved by maximising an objective function in which the modes of movement are inversely coded for two putative groups, with all values calculated at all possible nodes.

The proposed objective-function approach may be useful for identifying a node that best separates two predefined locomotor groups. However, in the present study, we aimed not only to locate a possible boundary between forward- and sideways-moving lineages, but also to reconstruct the evolutionary history of locomotor transitions under an explicit phylogenetic model.

For this reason, we used standard ancestral state reconstruction and stochastic character mapping rather than maximizing an ad hoc objective function across possible nodes. This approach allowed us to compare alternative transition-rate models (ER and ARD), estimate uncertainty in ancestral states at internal nodes, and quantify the posterior distribution of gains and reversals. We therefore retained the current phylogenetic framework, as it provides a model-based and more informative reconstruction of locomotor evolution across true crabs.

(3) Unfortunately, I find that the authors did not sufficiently discuss differences in the ecological niches of species with forward vs. sideways locomotion modes (including challenges of locomotion and substrate).

Likewise, what are the anatomic correlates of forward vs. sideways locomotion? For instance, how are the advantages assumed for sideways movement associated with a flattened body? Is it possible that the mode of motion is secondary to flattened/narrow body structure, which basically limits the distance between legs and thus makes the forward movement difficult - under this logic, the mode of movement would be a secondary phenomenon to body shape traits. How can one differentiate between this alternative and the one that puts the mode of movement in the centre of the story? On a related note, how do different modes of movement relate to the ability to fit into tight spaces - how does it relate to differences in leg joints?

Is it possible that the sideways movement maximises the scanned visual field per unit time/displacement, which may be beneficial for mostly forward-moving predators?

We thank the reviewer for this helpful comment. We agree that the previous version did not sufficiently address the possible relationship between locomotor mode and body shape, especially the alternative explanation that sideways locomotion may be secondary to carapace flattening. In response, we added a new morphological analysis using two carapace shape indices: relative carapace length (CL/CW) and relative carapace depth (CD/CS) (Methods, lines 178–185). In the revised Results, we report that relative carapace length differed significantly between forward- and sideways-moving taxa (phylogenetically informed ANOVA: F = 26.90, p < 0.001), whereas relative carapace depth did not differ significantly between the two groups (F = 1.18, p = 0.403) (Results, lines 209–214; Fig. S3). We also added this interpretation to the Discussion, noting that locomotor mode is associated with some aspects of carapace shape but is not explained by simple carapace flattening alone (Discussion, lines 318–325).

We also revised the Discussion to address the reviewer’s suggestions about possible functional advantages of sideways locomotion beyond rapid bidirectional escape. Specifically, we now mention that other possible advantages may include movement through confined spaces and visual-field sampling during locomotion (Discussion, lines 310-318).

Finally, we retained the existing discussion of ecological specializations in forward-moving lineages, including coordinated collective movement in soldier crabs, decoration and concealment in majoid crabs, and life inside confined host spaces in pea crabs. This discussion supports the broader point that the adaptive value of sideways locomotion may depend on ecological context.

(4) It is really difficult to decipher the information contained in the nodes (circles) in the printed black-and-white version of the manuscript.

We have changed the color scheme and strengthened the outlines of the node pie charts so that the ancestral-state probabilities can be more easily distinguished (Fig. 5). We also applied the same revised color scheme to Figure 3 and Figure S4 for consistency across the manuscript.

(5) Briefly, although I find the study interesting, the presented complexity may not be necessary given the endpoints; it can be achieved much more simply. Furthermore, the degree to which the conceptual analysis of different modes of locomotion was exercised was limited. The general approach may serve as a good model for the evolutionary analysis of other traits. The demonstration of traceability of the relations in question is a major contribution of the work.

We thank the reviewer for this constructive summary and for recognizing the broader value of our approach. We have addressed the methodological and conceptual points raised here in our responses to the specific comments above.

Strengths:

The research question and the novel combination of different data types.

We thank the reviewer for highlighting the research question and the novel combination of different data types as strengths of the study. We have revised the manuscript to further strengthen this integrative framework.

Weaknesses:

The complexity of the methods used, along with a limited discussion of the potential dynamics that may underlie the evolution of the sideways movement mode.

We have addressed these concerns in our responses to the specific comments above, particularly by clarifying the rationale for the behavioral quantification and expanding the discussion of morphology, ecological context, and functional hypotheses.

**Recommendations for the authors:**

**Reviewer #1 (Recommendations for the authors):**

(1) Unbiased analysis of angle distribution. The authors already extract continuous bout angles prior to binning. I recommend using these distributions directly to assess modality at the species level (e.g., unimodal vs bimodal, peak locations, mixture weights) before collapsing behavior into the Forward-Sideways Index. Even a simple circular density estimate or mixture model would clarify whether species classified as "forward" or "sideways" are behaviorally pure or mixed, and would provide a quantitative basis for claims about intermediacy.

We added mixture-model analyses of the continuous bout-angle distributions, including modality, peak locations, and mixture weights (Results, lines 196-204; Table S2).

(2) Justify or stress-test the 60° cutoff. The manuscript should either provide a clear biological or data-driven justification for using 60° as the boundary between forward and sideways bouts, or demonstrate that the main conclusions are robust to reasonable alternative cutoffs (e.g., 45°, 75°). A brief sensitivity analysis in the supplement would be sufficient and would greatly strengthen confidence in the classification. Alternatively (my preference) would be to let the data inform the cutoff.

We clarified the rationale for the 60° sector definition used to calculate FSI (Methods, lines 125-130; Fig. 2) and added an independent data-driven boundary analysis based on dominant peak locations, which yielded the same forward/sideways classification (Results, lines 204-208; Fig. S2).

(3) Sampling justification. I recommend explicitly acknowledging that sampling a single individual per species limits the ability to detect ontogenetic, size-dependent, or allometric variation in locomotor strategy. If feasible, adding even limited replication across size classes or individuals for a small subset of taxa (particularly those near the classification boundary) would substantially strengthen the conclusions; otherwise, the manuscript should more clearly delimit which claims do and do not rely on the assumption of within-species invariance.

We clarified that our conclusions concern broad interspecific patterns of predominant locomotor direction, rather than the full range of within-species variation (Methods, lines 102-108).

(4) I would suggest toning down or reframing statements about "no intermediates". If additional analyses are not added, I recommend revising statements that imply a strict absence of intermediates to language that reflects what is directly shown (e.g., bimodality in an index derived from binned data). This would better align the claims with the current evidence.

We revised the manuscript to avoid implying a strict absence of intermediates and now acknowledge that some taxa show mixed directional tendencies (Abstract, lines 27-29; Results, lines 191-208).

(5) Framing and claims about diversification. The discussion of sideways locomotion as a key innovation would benefit from clearer separation between observed correlations and causal inference. If trait-dependent diversification analyses are not added, I suggest consistently framing this section as a hypothesis supported by comparative patterns rather than a demonstrated mechanism.

We revised the Discussion to more clearly frame sideways locomotion as a possible key innovation associated with diversification, rather than as a demonstrated causal mechanism (Abstract, lines 31-34; Discussion, lines 294-309).
